# Microalgae as Novel Food Resources: Technological Breakthroughs, Application Bottlenecks, and Future Pathways

**DOI:** 10.3390/foods15122241

**Published:** 2026-06-22

**Authors:** Xiaomei Zhang, Weixian Chen, Hui Chen

**Affiliations:** 1 School of Food and Health Engineering, Zhengzhou University of Technology, Zhengzhou 450044, China; 20151013@zzut.edu.cn; 2 Institute of Systems, Molecular and Integrative Biology, University of Liverpool, Liverpool L69 7ZB, UK; weixian.chen@liverpool.ac.uk; 3 State Key Laboratory of Crop Stress Adaptation and Improvement, School of Life Sciences, Henan University, Kaifeng 475004, China; 4 Henan Key Laboratory of Synthetic Biology and Biomanufacturing, Henan University, Kaifeng 475004, China

**Keywords:** microalgae, novel food, sensory acceptability, production cost, regulatory approval, alternative protein

## Abstract

Global population growth and the demand for sustainable food systems have pushed microalgae into the spotlight as promising novel food resources. They are rich in protein, omega-3 fatty acids, and bioactive pigments including astaxanthin and phycocyanin. Unlike conventional farming, microalgae cultivation can be conducted on non-arable land and may reduce direct competition with conventional food crops for land resources, depending on the production system used. Regulatory progress in China, the European Union (EU), and the United States has resulted in the authorization or approval of several microalgal species and microalgae-derived ingredients for specific food and nutritional applications, including dietary supplements, infant nutrition products, and alternative protein ingredients. Despite these advances, broader commercial adoption remains constrained by several challenges, such as off-flavors and the dark green color, high production costs from closed photobioreactors and energy-intensive downstream purification, fragmented regulatory frameworks across jurisdictions and limited long-term data on bioavailability, allergenicity, safety, and dose–response relationships for some emerging strains. This review focuses on microalgae as novel food resources, covering regulatory approvals, strain selection, high-value utilization, and market translation, synthesizes evidence on nutritional evaluation, application scenarios, and global regulatory differences, analyzes key bottlenecks, and proposes pathways to bridge fundamental research with industrial practice. It also highlights unresolved knowledge gaps to guide future research and policy.

## 1. Introduction

According to United Nations projections, the global population is expected to continue growing over the next 50–60 years, peaking at approximately 10.3 billion around the mid-2080s [1]. Alongside population growth and increasing consumer demand for better health, precision nutrition and functional foods have become central to the modern food industry. Meanwhile, traditional food production systems are confronted with mounting pressures, including limited arable land, high water consumption, significant greenhouse gas emissions, and a widening protein supply gap. It is imperative to develop a sustainable food system with a reduced environmental footprint [2,3,4].

Among diverse candidate novel food resources, microalgae have become a strategic focus in the global future food landscape due to their unique biosynthetic advantages. Microalgae are photosynthetic autotrophic microorganisms widely distributed in freshwater and marine habitats. They offer several advantages including high photosynthetic efficiency, short growth cycles, adaptability to cultivation on saline or non-arable land, and no competition with conventional food crops for resources [5,6,7,8]. These characteristics have led to growing interest in microalgae as a potential source of proteins and other food ingredients. However, their actual contribution to protein security and environmental sustainability depends on species selection, production systems, and scale of implementation [9,10]. In the initial stages of microalgae bioresource utilization research, microalgae were largely regarded as a potential resource, with research and applications confined mainly to laboratories and small-scale production of nutraceuticals [11,12,13]. However, the worldwide microalgae market has experienced substantial growth in recent years. Major markets such as the European Union, the United States, and China have successively approved various microalgae-derived novel food ingredients, including *Euglena gracilis*, oil from *Microchloropsis gaditana*, (*M. gaditana*) and *Chlamydomonas reinhardtii* [14,15]. Microalgae are increasingly being evaluated and adopted for commercial food applications under relevant regulatory frameworks. Their application scope has expanded beyond traditional dietary supplements to a wider range of food categories, such as plant-based meat analogues, functional meal replacements, and foods for special medical purposes [16].

Extensive scientific research indicates that many microalgal species possess high nutritional value and contain a variety of bioactive compounds [17,18,19]. In terms of basic nutritional properties, the crude protein content of microalgae varies widely from 6% to 70% on a dry-weight basis, and most species exceed 40% dry weight [20]. The known microalgae species: *Arthospira maxima*, *Limnospira maxima*, and *Chlorella vulgaris* have the highest protein content, up to 60–70% of dry weight, providing all essential amino acids, with an amino acid score close to the Food and Agriculture Organization of the United Nations/World Health Organization (FAO/WHO) recommended standard [20,21,22,23]. However, protein quality assessment increasingly relies on digestibility-adjusted indices. While earlier studies frequently employed amino acid scores and PDCAAS, the FAO currently recommends the Digestible Indispensable Amino Acid Score (DIAAS) as a more accurate measure of protein quality. At present, DIAAS data are available for only a limited number of microalgal species, representing an important research gap. Protein digestibility varies substantially among microalgae and is influenced by species-specific cell-wall structures and processing technologies. *Limnospira* sp. generally exhibit high digestibility because they lack a rigid cellulose-rich cell wall, whereas species such as *Chlorella vulgaris* may show lower digestibility unless subjected to cell-disruption treatments. Although digestibility data have been reported for additional species, including *Tetraselmis* and *Microchloropsis*, comprehensive standardized in vivo evaluations remain scarce. Consequently, further research is required to establish robust DIAAS values and enable reliable comparisons with conventional protein sources [24].

In terms of functional bioactivities, microalgae are rich in omega-3 polyunsaturated fatty acids (EPA and DHA), natural antioxidant pigments such as astaxanthin, phycocyanin, and lutein, as well as functional polysaccharides (e.g., from *Limnospira* and *Chlorella*), along with abundant vitamins and minerals [24]. The mechanisms of microalgae-derived bioactive compounds such as fucoxanthin and phycocyanin in metabolic regulation, antioxidant activity, immune-related responses, and inflammatory pathways have been investigated in cellular, animal, and, to a more limited extent, human studies. These findings suggest potential applications in functional food development, although further clinical validation is required for many proposed health benefits [25,26].

This review critically examines microalgae as novel food resources, covering regulatory approvals, strain selection, high-value utilization, and market translation. We synthesize evidence on nutritional evaluation, application scenarios, and global regulatory differences, and analyze key bottlenecks. We then propose pathways to overcome these challenges, bridging fundamental research with industrial-scale practice, and highlight unresolved knowledge gaps alongside a comparative analysis with other alternative protein sources to inform future research and policy.

## 2. Methodology

An assessment of the scientific literature was carried out to ascertain the trend of publications related to microalgae as novel food resources. Relevant peer-reviewed journal articles published in English were retrieved from major bibliographic databases such as Web of Science, Scopus, PubMed, and ScienceDirect. The combinations of keywords used for the search included “microalgae food, microalgae-based foods, edible microalgae, alternative protein, functional food, novel food, bioactive compounds, food applications, food safety, and microalgae commercialization. studies examining microalgae as a direct food ingredient or finished food product, including safety, techno-functionality, consumer acceptance, or market analysis were also considered. The search was limited to articles published within the last 10 years to capture recent developments in the field. Additionally, the reference lists of selected articles were screened to exclude duplicate records, conference abstracts, non-English articles, and studies not directly related to food applications of microalgae. Overall, the focus of the search was to gather comprehensive information on the current state of research and development in utilizing microalgae in food products. This approach allowed for a thorough analysis of the potential benefits and challenges associated with incorporating microalgae into various food applications.

## 3. Current Status and Developments of Microalgae as Emerging Novel Food Resources

### 3.1. Developments of Microalgae as Novel Food Resources

Microalgae is an emerging resource for food due to its high nutritional value and potential for sustainable production. Different species such as *Arthrospira* sp., *Chlorella* sp., *Dunaliella* sp., *Microchloropsis* sp., and *Schizochytrium* sp. have been explored for application in various food products, including supplements, functional foods, and even meat substitutes. These microalgae are rich in proteins, essential fatty acids, vitamins, and antioxidants, making them a promising ingredient for enhancing the nutritional profile of food products [27]. The commercial potential of microalgae as a food source is steadily increasing, but with more focus towards dietary supplements, powders, tablets, and functional food ingredients rather than direct replacements for traditional food sources [28]. The market value of species such as *Arthrospira* sp. and *Chlorella* sp. has been steadily rising due to their recognized health benefits and versatility in various food applications [29]. Biomass and proteins extracted from microalgae are incorporated into a wide range of products such as bakery products, plant-based meat alternatives, and beverages to boost their nutritional content [30]. These developments of microalgae-based food are driven by technological advancements that allow for efficient cultivation and extraction processes, making them more cost-effective and scalable for commercial production [31]. These advances include improvements in cultivation systems such as closed photobioreactors and optimized open-pond systems that have enhanced biomass productivity and reduced contamination risks [32]. Furthermore, innovative downstream processing technologies such as enzymatic hydrolysis, pulsed electric fields, bead milling, ultrasound-assisted extraction, and membrane filtration have improved the recovery and functionality of proteins, lipids, and pigments from microalgal biomass. These technologies have increased protein digestibility and expanded the range of food applications for microalgae-derived ingredients [33]. This technology has also enabled the improvement of the functional properties of microalgae-derived ingredients, making them more suitable for use in a variety of food products [34]. For example, protein isolates and concentrates are now easily used to provide desirable emulsification, foaming, and gelling properties in food formulations [35], whereas microalgae-derived compounds such as phycocyanin, astaxanthin, β-carotene, and omega-3 oils are increasingly used as natural colorants, antioxidants, and nutraceutical ingredients. These developments align with growing consumer demand for clean-label and plant-based products [36]. But despite these advancements, challenges still remain in scaling up production and reducing costs to make microalgae-derived ingredients more competitive with traditional sources. The high production cost results from the energy-intensive cultivation and processing requirements of microalgae resources. Regulatory frameworks also limit the widespread adoption of microalgae-derived ingredients in the food industry because regulatory approval varies between countries. For example, as of July 2025, nine microalgae-related ingredients had been approved by the National Health Commission (NHC) as novel food ingredients in China, including both microalgal biomass products and algal oils (Table 1). Separately, *Limnospira platensis* and *Limnospira maxima* are listed as conventional food ingredients and are therefore not included in the total number of approved novel food ingredients. Consequently, China has approved eleven microalgae-related food ingredients in total, comprising nine novel food ingredients and two conventional food ingredients, whereas the European Union (EU) Regulation 2015/2283 establishes a centralized authorization system for novel foods, requiring a scientific safety assessment by the European Food Safety Authority (EFSA) and inclusion in the Union List before commercialization. Although several microalgal species and derived ingredients have been authorized in the EU, as shown in Table 2, many emerging microalgae products must still undergo a time-consuming and resource-intensive approval process, which can slow market entry and increase development costs [37,38]. Although the U.S. Food and Drug Administration (FDA), through a dual-track system of Generally Recognized as Safe (GRAS) notifications and food additive petitions, has placed some microalgae-related novel food ingredients on its safety lists, permitting their use as food ingredients and dietary supplements as shown in Table 3 [39]. These differences in regulatory requirements between jurisdictions create additional barriers to international commercialization and large-scale adoption of microalgae-derived food ingredients [40].

### 3.2. Techno-Functional Properties of Microalgae as Food Ingredients

Microalgae possess a wide range of functional properties that make them valuable ingredients in modern food systems beyond their nutritional benefits as shown in Table 4. These properties, which include antioxidant property, antimicrobial property, and emulsifying capabilities, are critical in influencing the behavior of food products during processing and storage. This functional versatility of microalgae is attributed to their unique protein composition, polysaccharides, pigments, and bioactive compounds. Microalgae also have the potential to act as a multifunctional ingredients capable of enhancing both technological performance and nutritional value in food formulations [16]. For example, microalgae species such as *Arthrospira platensis*, *Chlorella vulgaris*, and *Microchloropsis* spp. contain high levels of protein that sometimes exceed 50–70% of dry biomass. These proteins are not only a rich source of essential amino acids but also possess functional properties such as desirable solubility, emulsifying, foaming, and gel-forming capacities that contribute to the texture, stability, and sensory attributes of food products. Protein solubility is particularly significant because it influences other functional characteristics such as emulsification and gel formation in food products [41]. The emulsifying properties of microalgal proteins enable the stabilization of oil–water mixtures, which is essential in food products such as beverages, dressings, sauces, and dairy alternatives. Their amphiphilic molecular structure allows proteins to adsorb at oil–water interfaces, leading to the reduction in surface tension that improves emulsion stability. Similarly, their foaming properties contribute to the formation and stabilization of air bubbles in foods such as whipped products, bakery items, and dessert formulations. These characteristics position microalgae as potential natural alternatives to synthetic emulsifiers and stabilizers, supporting the growing demand for clean-label products [42]. Microalgae also possess significant water and oil-holding capacities, which are critical for maintaining moisture, texture, and mouthfeel in processed foods. These properties are particularly valuable in plant-based meat analogues, bakery products, and dairy substitutes, where ingredient functionality directly influences consumer acceptance. Furthermore, certain microalgal proteins can form gels under specific conditions, contributing to structural integrity and desirable textural characteristics in food systems [43].

In addition to their technological functions, microalgae serve as sources of natural functional ingredients. For example, astaxanthin is a remarkably potent, naturally occurring carotenoid derived primarily from the microalga *Haematococcus pluvialis*. It is known for its powerful antioxidant properties due to its unique molecular structure, which allows it to physically span the entire cellular membrane and protect cells from oxidative damage through its ability to neutralize free radicals [44]. Hecht et al. [45] performed an 84-day randomized, double-blind, placebo-controlled study and demonstrated that daily supplementation with 4 mg of *H. pluvialis* astaxanthin significantly alleviated chronic and acute eye fatigue symptoms in developing eyes of schoolchildren aged 10–14 years. Meanwhile, such supplementation improved tear film stability and sustained ocular surface health. In the field of skin health, Sekikawa et al. [46] conducted an 8-week double-blind, randomized controlled trial. They first confirmed that daily oral intake of 6 mg of *H. pluvialis* astaxanthin significantly increased the minimal erythema dose (MED) required to induce visible erythema in adult skin and subjectively improved hair dryness (*p* = 0.025). Dose–response analysis by Sekikawa et al. [47] in a randomized, placebo-controlled, double-blind trial investigated whether daily supplementation with 9 mg/day of astaxanthin and 50 mg/day of tocotrienols for six weeks could improve ocular function in healthy adults. The study reveals that the combination of astaxanthin and tocotrienols may help protect against the temporary deterioration in visual function caused by prolonged screen use, particularly by preserving visual acuity and functional visual acuity in middle-aged and older adults [47], whereas Yoshida et al. [48] performed a randomized, double-blind, placebo-controlled trial involving 61 non-obese adults (25–60 years old) with mild hypertriglyceridemia, with each participant receiving 0, 6, 12, or 18 mg/day of natural astaxanthin for 12 weeks. The study showed that natural astaxanthin supplementation for 12 weeks improved key lipid markers by lowering triglycerides, raising HDL cholesterol, and increasing adiponectin levels in people with mild hyperlipidemia. Beyond human health, Zhang et al. [49] investigated whether astaxanthin-rich *H. pluvialis*, a microalga rich in natural astaxanthin, could protect myofibrillar proteins (MPs) in meat from oxidative damage and improve their gel-forming properties, which are critical for meat texture, water retention, and overall product quality. The study showed that astaxanthin-rich *H. pluvialis* is a promising natural antioxidant ingredient for meat processing because it reduces protein oxidation and restores gel functionality, which can improve texture, moisture retention, and overall quality of meat products exposed to oxidative stress.

Phycocyanin is another pigment found in microalgae that serves as a source of natural functional ingredients. It is a blue pigment that has antioxidant and anti-inflammatory properties due to its unique chromoprotein structure, specifically the covalently bound linear tetrapyrrole called phycocyanobilin. This pigment is commonly used in food and cosmetic industries for its health benefits and vibrant color, making it a popular choice for natural products seeking to enhance their nutritional value and visual appeal. The immunomodulatory and anti-inflammatory activities of phycocyanin peptides derived from the cyanobacterial pigment phycocyanin were investigated by Liu et al. [50] which demonstrated that phycocyanin peptides mediated the induction of heme oxygenase-1 (HO-1) expression through the Keap1-Nrf2-HO-1 pathway, which reduced oxidative stress, inflammation, and fibrotic changes associated with pulmonary fibrosis in cell-based models. Thus, it is suggested that phycocyanin peptides could serve as a promising natural therapeutic candidate for pulmonary fibrosis, although animal and human studies are needed to confirm efficacy and bioavailability. Jiang et al. [51] also investigated the anti-cancer activity of C-phycocyanin (C-PC) from *Spirulina platensis* in MDA-MB-231 triple-negative breast cancer (TNBC) cells. The study revealed that C-PC significantly inhibited the proliferation and induced apoptosis of MDA-MB-231 cells through the inhibition of cell proliferation and colony formation. C-PC also induced G0/G1 cell-cycle arrest by decreasing Cyclin D1 and CDK2 while increasing p21 and p27 and promoting apoptosis through activation of the Fas/caspase-3 death receptor pathway. C-PC also reduced cancer cell migration by suppressing COX-2 expression.

Polysaccharides obtained from microalgae act as prebiotics by resisting digestion in the upper gastrointestinal tract and reaching the colon intact, where they are selectively fermented by gut microbiota [52]. This alludes to the fact that microalgae polysaccharides are characterized by unique glycosidic linkages such as β-(1→3) and β-(1→4) bonds, which resist hydrolysis by human digestive enzymes [53]. Although their composition varies among different species of microalgae, these polysaccharides have shown promising potential in promoting gut health and modulating the gut microbiota composition. Lv et al. [54] investigated whether polysaccharides extracted from the microalga *Chlorella pyrenoidosa* could function as a prebiotic by resisting digestion and selectively promoting beneficial gut microbes during fermentation in simulated saliva, gastric, and small-intestinal digestion models. The study showed that polysaccharides exhibit clear prebiotic potential because (i) they survive digestion in the upper gut, (ii) they are selectively fermented by colonic microbiota, (iii) they promote beneficial bacterial populations (especially *Parabacteroides distasonis*), (iv) they suppress less desirable microbes, and (v) they increase health-promoting short-chain fatty acids.

**Table 4:** Bioactive compounds produced by microalgae and health benefits.

Microalgal Source	Bioactive Compound	Proposed Health Effect	Type of Evidence	Human Dose	References
*Haematococcus pluvialis*	Astaxanthin	Reduction in oxidative stress and also supports skin vitality, eye health, cardiovascular function, and athletic recovery	Multiple human clinical trials, supported by mechanistic studies	4–12 mg/day	[55]
*Schizochytrium* spp.	DHA (Docosahexaenoic acid)	Brain, visual development, and cardiovascular health	Extensive human clinical evidence and have been approved as food and supplement ingredient	200–1000 mg/day DHA	[56]
*Microchloropsis* spp., *Phaeodactylum tricornutum*, *Odontella aurita*	EPA (Eicosapentaenoic acid)	Cardiovascular protection, triglyceride reduction, and anti-inflammatory effects	Extensive human evidence for EPA	250–2000 mg/day EPA	[57,58]
*Arthrospira platensis*	Phycocyanin-rich biomass	Improved lipid profile, reduced oxidative stress, and inflammation	Human intervention studies using Spirulina biomass; mechanistic evidence for phycocyanin	1–8 g/day Spirulina biomass	[59,60]
*Chlorella vulgaris*	Carotenoids, polysaccharides and related bioactive fractions (whole biomass)	Improved antioxidant status, blood pressure regulation and immune support	Human supplementation studies, though heterogeneous	3–10 g/day Chlorella biomass	[61,62,63]
*Dunaliella salina*	β-Carotene	Provides provitamin A activity, enhanced antioxidant and eye health	Strong human evidence for β-carotene, but limited microalgae-specific clinical trials	6–30 mg/day β-carotene	[64,65]
*Dunaliella salina*, *Chlorella* spp.	Lutein	Support of macular and retinal health	Human evidence for lutein supplementation but fewer microalgae-specific clinical studies	10–20 mg/day lutein	[66]

### 3.3. Safety Evaluation and Regulatory Landscape of Microalgae-Derived Bioactive Compounds

The successful commercialization of microalgae-derived products depends on safety assessments and compliance with increasingly complex regulatory frameworks. This regulatory framework includes guidelines for the cultivation, processing, and marketing of microalgae products to ensure consumer safety and environmental sustainability. This framework varies across different countries and regions, particularly regarding approval pathways, daily intake limits, and labeling requirements, creating challenges for global market expansion [38]. The safety evaluation of microalgae-derived bioactive compounds generally focuses on toxicological characterization, allergenicity, nutritional impact, contaminant levels, and estimated dietary exposure. Particular attention is also given to the accumulation of heavy metals, marine toxins, excessive iodine, and other undesirable substances that may arise during cultivation and processing. Regulatory agencies also assess the stability, purity, manufacturing processes, and intended conditions of use of novel ingredients. The EU operates the most precautionary regulatory systems under the regulation of microalgae-derived bioactive compounds (EU 2015/2283), in which products that were not consumed to a significant degree before May 1997 are classified as novel foods and require pre-market authorization following scientific evaluation by the EFSA. The assessment includes toxicological studies, exposure analyses, nutritional evaluation, and proposed use conditions. Approved products are subsequently listed in the Union List of Novel Foods, often with specific restrictions regarding dosage, target populations, and labeling requirements, as shown in Table 2. This approach emphasizes consumer protection but can significantly increase the time and cost required for commercialization [37]. For example, a scientific risk assessment by the European Food Safety Authority (EFSA) on the evaluation of whether an ethanolic extract of the microalga *Phaeodactylum tricornutum* can be approved as a novel food supplement in the EU. The overall conclusion was that the ethanolic extract of *Phaeodactylum tricornutum* could not be considered safe as a novel food supplement based on the key evidence, which includes insufficient demonstration of a consistently safe production process, incomplete compositional characterization, lack of a history of safe human consumption, adverse findings in animal toxicity studies, and unresolved concerns regarding phototoxic chlorophyll-derived compounds [67]. Also, the assessment of the safety of a DHA-rich oil derived from the microalga *Schizochytrium limacinum* (strain FCC-3204) as a novel food supplement through the increase of the maximum daily intake from 250 mg of DHA/day to 3 g of DHA/day for adults by EFSA got partial approval of safety. This is because the assessment by EFSA concluded that the algal oil is safe as a food supplement providing up to 1 g of DHA/day, and the approved target population was adults but excluding pregnant and lactating women [68]. The distinguishing feature of the EU framework and others is the establishment of scientifically derived intake limits. For example, EFSA’s evaluation of the suitability of astaxanthin, a carotenoid derived primarily from the microalga *Haematococcus pluvialis*, concluded that a combined intake of up to 8 mg/day from food supplements and dietary sources is safe for adults, while younger populations may exceed acceptable exposure levels. EFSA further established an acceptable daily intake (ADI) of 0.2 mg/kg body weight for astaxanthin [69]. Similarly, novel DHA-rich oils derived from *Schizochytrium limacinum* have been authorized with specific conditions of use and maximum intake levels based on exposure assessments. Such quantitative intake restrictions are characteristic of the EU’s risk-based regulatory philosophy [68]. In contrast, the US employs a comparatively flexible and industry-driven regulatory approach. Many microalgae-derived ingredients intended for food use are marketed under the GRAS framework administered by the FDA. This system allows manufacturers to demonstrate the safety of ingredients for their intended use by submitting a GRAS notice to the FDA. Examples of microalgal ingredients that have been granted GRAS status include *Arthrospira* and DHA-rich algal oils from *Schizochytrium*. Although manufacturers can choose to withdraw their GRAS notification at any time if new information arises that may affect the safety of the ingredient. This flexibility allows for continuous evaluation and adaptation to ensure consumer safety. For example, the GRAS notice of *Arthrospira* biomass (GRN 391) was withdrawn by the notifier prior to FDA evaluation, whereas numerous GRAS notices have been successfully submitted for *Schizochytrium*-derived oils, including GRN 137 and subsequent notices covering specific strains and product specifications. Also, unlike the EU, the US generally does not establish mandatory, population-wide upper intake limits for most GRAS ingredients. Instead, acceptable use levels are determined on a product-specific basis according to the available safety data and intended applications described in individual GRAS notices. This framework facilitates innovation and market entry but places substantial responsibility on manufacturers to ensure safety and regulatory compliance [70]. The regulatory management of food ingredients in China is complex and stringent, with strict guidelines set by the China Food and Drug Administration (CFDA) to ensure food safety and quality. Companies looking to introduce new ingredients or products into the Chinese market must navigate a thorough approval process that includes safety assessments and registration requirements. The National Health Commission (NHC) is responsible for safety review and approval of new food raw materials and related novel food ingredients, while enforcement and market supervision are supported by the State Administration for Market Regulation (SAMR) [71,72]. Under Chinese food regulation, ingredients that lack a history of consumption in China are generally classified as new food raw materials and are subject to pre-market safety evaluation and approval before commercialization [73]. Traditionally consumed microalgae such as *Limnospira* sp. are listed as conventional food ingredients and are commonly used in food and health food applications. In contrast, *Chlorella* sp., while widely used in food and health food products, was formally approved as a new food raw material (No. 19, 2012) and is therefore not classified as a conventional ingredient. Other novel microalgal ingredients similarly require regulatory assessment as new food raw materials depending on their intended use and production process, as shown in Table 1 [74].

Regulatory requirements for novel food ingredients differ across jurisdictions, with some countries having more stringent requirements than others. For example, in EU, novel foods are regulated under Regulation (EU) 2015/2283, which requires pre-market safety assessment and authorization by the European Commission following scientific evaluation by the EFSA [75]. The EFSA evaluation of the safety of algal meal from the microalga *H. pluvialis* containing astaxanthin as a novel food under Regulation (EU) 2015/2283 sought to extend its use beyond food supplements into dairy alternatives, beverage whiteners, and fruit juices. The evaluation concludes that the algal meal is safe for the proposed new uses in dairy analogs and fruit juices provided that children and adolescents do not consume astaxanthin-containing food supplements on the same day as foods containing the novel ingredient [76]. This shows that in the EU health claims are regulated under Regulation (EC) No. 1924/2006, which requires scientific substantiation and pre-authorization before use on food labels [77], whereas in the US, food and dietary supplement products are regulated under FDA labeling and dietary supplement regulations, which require ingredient labeling, nutrition information, and compliance with rules governing health and structure/function claims. Structure/function claims are permitted under specific conditions provided they are truthful and not misleading, while disease-related health claims are subject to FDA authorization and scientific substantiation requirements [78]. In China, food labeling and health food regulation are governed under the national food safety framework, with requirements for ingredient identification, nutritional labeling, and regulatory control over health food claims through approval or filing systems administered by national authorities [79].

### 3.4. Advances in Microalgae Processing, Protein Texturing, and Precision Nutrition Formulations

Conventional microalgal biomass products often face challenges associated with variable bioactive compound concentrations, limited digestibility due to recalcitrant cell walls, and physical or chemical characteristics that may restrict their incorporation into food products. Consequently, recent advances in microalgae processing have focused on developing innovative techniques to enhance the extraction of bioactive compounds, improve digestibility through cell wall disruption methods, and modify physical or chemical properties to increase their functionality in food applications. These advancements allow for the production of standardized ingredients enriched in specific bioactive compounds, proteins, lipids, and pigments rather than the direct use of whole biomass [80]. Modern processing technologies such as ultrasound-assisted extraction, pressurized liquid extraction, enzyme-assisted extraction, supercritical CO_2_ extraction, membrane filtration, and chromatographic purification have improved the recovery and purification of valuable compounds such as phycocyanin, carotenoids, and omega-3 fatty acids while reducing solvent consumption and improving process efficiency. These technological advances are facilitating the development of high-quality microalgal ingredients with improved consistency, functionality, and applicability in functional foods and nutraceuticals [81].

The growing interest in precision nutrition has further stimulated the development of multi-component formulations designed to address specific nutritional requirements and health outcomes. Precision nutrition aims to tailor dietary interventions according to individual physiological characteristics, lifestyle factors, and health status. Within this context, microalgae represent attractive sources of proteins, omega-3 fatty acids, pigments, vitamins, minerals, and other bioactive compounds that can be incorporated into targeted nutritional formulations. For example, Deng et al. [82] elucidated at the molecular level that fucoxanthin and low-molecular-weight fucoidan components synergistically improve insulin resistance and enhance insulin sensitivity in mice with high-fat diet-induced metabolic syndrome, thereby verifying the promising potential as a combined functional food against metabolic syndrome. Although the efficacy of such formulations requires validation through well-designed human clinical studies. Thus, microalgae-derived ingredients offer promising opportunities for the development of personalized nutrition products aimed at supporting metabolic health, immune function, and gastrointestinal well-being [83]. Microalgal proteins are also an emerging alternative protein source owing to their high protein content, balanced amino acid composition, and potential for sustainable production. Many commercially relevant microalgal species contain all essential amino acids and can be cultivated on non-arable land with reduced competition for agricultural resources compared with conventional protein crops [84]. In addition to their nutritional value, microalgal proteins are frequently associated with naturally occurring pigments, antioxidants, vitamins, and minerals that may provide additional functional benefits in food applications. Table 5 presents a comparative analysis of microalgal proteins with soy/pea proteins, mycoprotein (e.g., Quorn), and fermentation-derived proteins (e.g., yeast), highlighting the potential advantages of microalgal proteins in terms of sustainability and nutritional content [28].

Advances in protein texturing technologies have expanded opportunities for utilizing microalgal proteins in next-generation food products. High-moisture extrusion is currently one of the most widely investigated technologies for producing fibrous structures that mimic the texture of animal meat. The incorporation of microalgal biomass into protein extrusion systems has demonstrated the feasibility of generating structured products suitable for meat analog applications, although formulation composition and processing conditions strongly influence texture development and product quality. Consequently, strategies such as biomass pretreatment, cell disruption, protein enrichment, and lipid modification are being investigated to improve the texturization performance of microalgal ingredients. Olivas et al. [85] investigated whether high-moisture extrusion (HME) could be used to produce nutritionally improved meat analogues from faba protein concentrate (FPC) combined with single-cell proteins (SCPs) from the microalga *Chlorella vulgaris* and the bacterium *Xanthobacter* spp. The study evaluated structure, texture, amino acid quality, digestibility, mineral bioaccessibility, and anti-nutritional factors. The study demonstrates that combining faba protein concentrate with bacterial and/or microalgal single-cell proteins and processing them via high-moisture extrusion can produce meat analogues that have convincing meat-like structure and texture, offer substantially improved amino acid profiles, maintain very high protein digestibility, contain reduced anti-nutritional factors, and show nutritional characteristics approaching those of animal meat [85].

Beyond extrusion, emerging technologies such as 3D food printing, high-pressure homogenization, ultrasonication, and shear-cell processing are being explored to create customized food structures and tailor sensory attributes. In particular, 3D food printing offers opportunities to precisely control ingredient distribution, texture, and nutritional composition, thereby supporting the development of personalized nutrition products for specific consumer groups. For example, Ha et al. [86] investigated whether the microalga *Auxenochlorella protothecoides* can be used as a sustainable food ink for 3D printing seafood analogs. The study shows that *Auxenochlorella protothecoides* can serve as a sustainable, highly printable food ink for 3D-printed seafood alternatives. A formulation containing 36% microalgae provided the best balance of flowability and shape retention, while printer settings such as nozzle diameter and infill density enabled the researchers to mimic the texture of multiple fish species without changing the recipe itself. This is a significant step toward customizable, sustainable, plant-based seafood produced through additive manufacturing [86]. Practical applications of microalgal proteins have expanded to include meat analogues, dairy products, fermented foods, baked goods, beverages, and nutritional supplements. But the commercial viability of microalgal proteins still faces challenges related to scalability, cost-effectiveness, and consumer acceptance. The presence of pigments and volatile compounds can contribute characteristic color, odor, and flavor attributes that may not align with consumer expectations. In particular, lipid oxidation products, aldehydes, ketones, sulfur-containing compounds, and other volatile metabolites have been associated with the distinctive sensory profiles of some microalgal ingredients. Consequently, flavor optimization, strain selection, fermentation-based processing, and ingredient purification are crucial steps in ensuring the market success of microalgal ingredients [87].

**Table 5:** Comparison of alternative protein sources.

Feature	Microalgae	Soy/Pea	Mycoprotein (e.g., Quorn)	Fermentation-Derived	References
Primary Source	Photosynthetic aquatic microorganisms	Legumes (soybeans, yellow peas)	Fungus (*Fusarium venenatum*) grown in bioreactors	Single-cell microorganisms (e.g., *Saccharomyces cerevisiae*, *Candida utilis*)	[88,89,90,91]
Protein Content (dry weight)	50–70%	35–50% (isolates: 80–90%)	45%	45–60%	[92,93,94]
Amino Acid Profile	Complete (all essential amino acids)	Soy: complete; Pea: near-complete (slightly low in methionine)	Complete (comparable to egg/milk)	Complete (contains all essential amino acids)	[41,95,96]
Key Nutrients	Iron, phycocyanin, omega-3 EPA/DHA (in some species).	Isoflavones (soy), fiber, iron, folate	High fiber (chitin/glucan), low fat, B vitamins, no cholesterol	B vitamins (especially B1, B2, B6), selenium, beta-glucans	[41,97,98,99]
PDCAAS (Protein Digestibility)	0.67–0.84 (spirulina); digestibility 70–90% depending on processing	Soy: 0.90–1.00; Pea: high-quality protein	Recognized as high-quality complete protein	Recognized as high-quality complete protein	[16,92]
Sustainability	Low land/water use, no arable land required	Legumes fix nitrogen; lower environmental impact than animal protein	Low greenhouse gas emissions, land, and water requirements	Fast growth, low land use, can utilize waste streams	[100,101,102]
Allergenicity	Low; rare cross-reactivity	Soy: common allergen; Pea: low-moderate	Rare; possible in individuals with mold allergy	Very low; rare yeast allergy	[103,104,105,106]
Processing Complexity	High (cultivation, harvesting, cell disruption, drying)	Moderate (cleaning, milling, extrusion, protein isolation)	Moderate-high (fermentation, RNA reduction, texturization)	Low-moderate (fermentation, harvesting, drying)	[107,108,109,110]

## 4. Barriers to Commercialization of Microalgae-Based Foods

### 4.1. Current Market Status and Commercialization Challenges

Commercially established microalgae-based products currently include dietary supplements derived from *Arthrospira* sp. and *Chlorella* sp.; omega-3-rich oils from *Schizochytrium* spp.; natural colorants such as phycocyanin; and ingredients incorporated into beverages, bakery products, dairy alternatives, and meat analogues. But despite increasing scientific and commercial interest, the penetration of microalgae into mainstream food markets remains limited. Most commercially successful applications are concentrated in high-value nutritional supplements and specialty ingredients rather than mass-market food products. This is because biomass prices are higher than traditional food ingredients, making it challenging for microalgae-based products to compete on price with conventional options. For example, the price of algal biomass cultivated in batch and semi-continuous approaches has been shown to range from $1130 to $2040 per metric ton [111]. Although, prices scale exponentially depending on processing constraints, cellular disruption, extraction of specific high-value pigments (like C-phycocyanin), and laboratory-grade purifications [112].

This limited market adoption reflects several interconnected barriers created by higher production costs, sensory challenges, regulatory complexity, and remaining scientific uncertainties regarding ingredient functionality and consumer acceptance [113]. Numerous studies have demonstrated that the incorporation of microalgal biomass into food products can enhance protein content and nutritional value [114]. However, the level of inclusion is frequently constrained by changes in flavor, color, texture, and overall consumer acceptability, which often become more pronounced as microalgae concentrations increase [87,113]. Consequently, while proof-of-concept applications have been demonstrated across a wide range of food categories, successful large-scale commercialization remains challenging [115].

### 4.2. Sensory Acceptability Bottlenecks

Among the factors limiting commercialization, sensory acceptability remains one of the most significant barriers. The incorporation of microalgae into foods may introduce undesirable color, aroma, flavor, and textural attributes that reduce consumer acceptance, particularly in products where consumers expect mild sensory characteristics [87]. Off-flavor formation may also occur during processing and storage of products containing microalgae because microalgae contain substantial amounts of polyunsaturated fatty acids, which are susceptible to oxidation and may produce aldehydes, ketones, alcohols, and other volatile compounds associated with grassy, marine, fishy, or rancid sensory notes [116]. The majority of consumers are able to identify microalgal off-flavors in products due to their distinct and often unpleasant volatile profiles, confirming the presence of sulfur-containing compounds and fatty acid-derived aldehydes such as hexanal. In addition to sulfur-containing compounds, terpenoid derivatives, and other volatile metabolites have been identified as contributors to the characteristic aroma profiles of certain microalgal species, highlighting the complexity of off-flavor formation in microalgae [117]. The intense green, blue-green, or brown pigmentation associated with chlorophylls, phycobiliproteins, and carotenoids can substantially alter the appearance of food products, limiting consumer acceptance in categories where conventional color expectations are well established. For example, Arouna et al. [118] compared three variants of *Chlorella vulgaris* flour, green (wild type, pigment-rich), honey (reduced chlorophyll, yellow carotenoids retained), and white (largely pigment-free) by examining how 24 h fermentation with lactic acid bacteria (LAB) and yeasts affected their biochemical composition and antioxidant properties. The study shows that while green *Chlorella* sp. naturally possesses the strongest antioxidant profile, white *Chlorella* sp., which is the most attractive for food applications because of its neutral color and flavor, can be substantially upgraded through fermentation. This makes pigment-deficient strains promising ingredients for functional foods, nutraceuticals, and alternative-protein products [118]. Various mitigation strategies have been investigated to increase the sensory acceptability of microalgae-based products. Among these, mitigation techniques include fermentation, encapsulation, flavor masking, ingredient purification, and process optimization. Fermentation has shown potential to modify volatile profiles and improve sensory properties; for example, lactic acid bacteria fermentation can degrade chlorophylls, reducing green color intensity while potentially increasing overall antioxidant capacity [119]. Encapsulation technologies have also been used to enhance the sensory acceptability of microalgae-based products through the reduction in flavor release and enhancing ingredient stability [120]. For example, encapsulation technologies have also been used to enhance the sensory acceptability of microalgae-based products through the reduction in flavor release and enhancing ingredient stability. For example, Djihad et al. investigated whether microencapsulation by complex coacervation could make the microalga *Chlorella vulgaris* a more acceptable ingredient in pear-based snacks by masking its undesirable sensory characteristics (strong taste, odor, and dark green color) while preserving its nutritional value. The study shows that encapsulating *Chlorella vulgaris* in pea protein carrageenan microcapsules substantially improved its stability, controlled release, and consumer acceptance in pear snacks, demonstrating a viable approach for creating more appealing algae-based functional foods [121]. Additionally, the inclusion of natural flavoring agents such as fennel, rosemary, and ginger can effectively mask the intense grassy, umami, or “fishy” off-flavors associated with microalgae biomass. However, no single approach has consistently eliminated sensory limitations without affecting cost, nutritional quality, or process complexity. As a result, sensory optimization remains a critical research priority for broader commercialization of microalgae-based foods [122].

### 4.3. Economic Constraints in Microalgae Production and Processing

The high cost of biomass production is a constraint limiting the commercialization of microalgae-based food products. This is because microalgae are typically cultivated in either open raceway ponds or closed photobioreactor systems, each of which presents distinct economic advantages and limitations. For example, open raceway ponds generally offer lower capital and operating costs and are widely used for large-scale production of species such as *Arthrospira* sp. and *Dunaliella* sp. Quantitative techno-economic analysis indicates that semi-continuous cultivation in open raceway ponds achieves a minimum biomass selling price (MBSP) of 1130–1200 per metric ton, while batch cultivation costs significantly more at 1380–2040 per metric ton. However, these systems are susceptible to contamination, environmental fluctuations, and variable productivity, which can affect product consistency and economic performance [111]. In contrast, closed photobioreactors provide greater control over cultivation conditions and may achieve higher biomass productivities compared to open systems. For example, Francke et al. evaluated a new floating tubular photobioreactor (PBR) that can be partially immersed in natural surface waters to regulate temperature without active cooling systems using the microalga *Tetradesmus obliquus* as a test organism. The study demonstrated that a floating, partially immersible tubular photobioreactor can use the thermal mass of natural water bodies for passive temperature control, maintaining suitable cultivation temperatures while achieving stable growth and high-quality biomass production that ranges between 161 mg L^−1^ day^−1^ and 230 mg L^−1^ day^−1^ [123]. Nevertheless, the substantial capital investment, energy demand, and operational complexity associated with photobioreactor systems significantly increase production costs, with harvesting and drying stages potentially representing the majority of the final product value [29].

The downstream processing of microalgae is also one of the most expensive stages of the production chain. This is because the processes of cell disruption, biomass harvesting, drying, extraction, fractionation, and purification are energy-intensive. They substantially contribute to about 15–90% of total metabolite production costs in industrial processes, depending on the biochemical composition of the target product [124]. The robust cell walls of many microalgal species further complicate the efficient recovery of proteins and bioactive compounds because they require additional processing steps. For example, Valdovinos-García et al. [125], in a simulation study of lipid production from the microalga *Chlorella vulgaris*, compared different harvesting and cell-disruption technologies. Bead milling showed the highest energy demand (8.79–8.88 kWh/kg lipids) and operating cost (USD 41.06–41.41/kg). High-pressure homogenization (HPH) achieved the lowest energy consumption (5.39–5.46 kWh/kg) and required the least freshwater, consuming 5.3 m^3^/kg lipids, while costing USD 34.26–34.71/kg. Pressing required 5.80–5.88 kWh/kg and had the lowest operating cost (USD 27.27–27.88/kg) [125]. Although advanced extraction technologies such as supercritical fluid extraction, enzyme-assisted extraction, membrane filtration, and chromatographic purification can improve product quality and purity (with enzyme-assisted and supercritical fluid extraction achieving yields up to 97%). Their implementation at industrial scale is limited by equipment costs, energy requirements, and process complexity [126].

### 4.4. Gaps in Scientific Understanding

There are notable gaps in both fundamental scientific research and public awareness regarding microalgae foods, compounded by disconnects along the industrial chain, further restricting industry development. At the scientific research level, the absorption and metabolism mechanisms, physiological function, long-term consumption safety, and dose–response relationships of some novel microalgae bioactive ingredients (e.g., novel algal polysaccharides, fucoxanthin, microalgal bioactive peptides) in humans are still unclear. Long-term human clinical trial data are still lacking, and some efficacy claims remain at the stage of in vitro cell and animal experiments, making it difficult to support product functional claims and large-scale commercial promotion. For example, fucoxanthin, an important carotenoid bioactive ingredient in microalgae, has demonstrated antioxidant and anti-inflammatory effects in in vitro and animal experiments [127,128], but data regarding its absorption and conversion rates in humans, tissue distribution, and safety with long-term consumption (over 12 months) remain scarce. Similar research gaps also remain in novel algal polysaccharides, which degradation pathways in the human body and mechanisms of interaction with gut microbiota are not fully elucidated, and most studies are limited to in vitro cell experiments, lacking large-sample, long-term human clinical trials. Another critical gap concerns the true ileal digestibility of microalgal proteins. When cell walls are not fully disrupted, digestibility can be significantly lower than the often-cited values, affecting protein quality scores and nutritional claims. Furthermore, potential unintended effects of gene-edited strains, such as off-target metabolites and environmental escape, require long-term ecological verification that has not yet been completed.

At the public awareness level, consumer acceptance of microalgae-derived foods remains limited in many markets. Studies investigating consumer attitudes toward algae-based foods have identified several barriers, including low familiarity with microalgae as food ingredients, food neophobia, concerns regarding sensory attributes such as color, odor, and taste, and limited awareness of their nutritional and environmental benefits [115,129,130]. In particular, sensory characteristics associated with some microalgal species, including marine-like, grassy, or fishy notes, may negatively influence consumer acceptance when incorporated at high levels into food products. Furthermore, consumers may have difficulty distinguishing edible microalgae approved for food use from harmful algal blooms or toxic algal species, leading to uncertainty regarding product safety. As a result, willingness to purchase microalgae-derived foods often remains lower than that for conventional food products, creating challenges for market penetration and commercialization. Improving consumer education, transparent communication of safety and nutritional benefits, and the development of products with optimized sensory properties will therefore be essential for broader market acceptance [131,132].

## 5. Future Directions and Pathways to Address Key Challenges

Addressing the multifaceted challenges facing microalgae as novel food resources requires a coordinated strategy spanning science communication, standardization, technological innovation, strain engineering, and circular economy integration.

Science communication and consumer education are essential to break down cognitive barriers and improve market acceptance. Drawing on clinical nutrition research and toxicological safety data, objective information about the nutritional value, health benefits, and low-carbon, sustainable advantages of microalgae-derived foods should be disseminated through popular science articles, promotions by health-focused key opinion leaders (KOLs), product experience events, and in-person tastings. This will help correct consumer misconceptions about off-flavor and safety. At the same time, collaboration with food companies is needed to optimize product formulations, sensory attributes, texture, and packaging. By leveraging advances in food processing technologies—particularly mild, efficient deodorization and color adjustment techniques, as well as precision flavor modification—sensory defects can be effectively mitigated. A diverse range of end products, including snacks, beverages, baked goods, and meal replacements, should be launched to align with consumer habits. Differentiated frameworks for raw materials, ingredients, and finished foods, along with global regulatory harmonization, will facilitate cross-border trade.

Standards and database development are important pillars for promoting the standardized development of the microalgae food industry. Public databases for component analysis, efficacy evaluation, and safety assessment should be established. International standards for microalgal protein purity, fucoxanthin detection, and other bioactive ingredients are urgently needed. China’s GB/T 31520-2015 [133] (determination of astaxanthin in *Haematococcus*—High performance liquid Chromatography method) provides a model. The EU is harmonizing standards to simplify novel algae food approvals. Species with aquaculture use history (e.g., *Microchloropsis)* could be fast-tracked using feed experience to replace toxicity testing. Simplifying approval procedures for third-country conventional microalgae would enrich EU markets.

Technological convergence and intelligent manufacturing are core drivers for scaling up the microalgae food industry. An intelligent integrated manufacturing system, covering precision breeding, intelligent cultivation, online monitoring, automated harvesting and green processing, is the key to realizing low-cost and large-scale production. Artificial intelligence, big data, and digital bioreactors technologies enable real-time regulation of light intensity, temperature, and nutrients, improving the yield and quality of microalgal biomass. Low-carbon modes (photovoltaic-PBR coupling, heterotrophic-autotrophic mixotrophy, raceway-closed reactor combinations) reduce energy and land occupation. Automation upgrading of harvesting and processing contributes to cost reduction. Food processing upgrading should focus on mild deodorization and color adjustment, and adopt the combined “enzymatic disruption + membrane separation” to increase extraction yield and minimize nutrient loss. Directed texture re-engineering and precision flavor modification will improve algae-derived alternative proteins. Stabilization and delivery technologies for bioactive ingredients can enhance bioavailability. Full-value-chain utilization of processing byproducts and circular processing models are needed.

Next-generation strain design is indispensable because synthetic biology, systems biology, and microbiome technologies could enable the development of ideal strains with high productivity, stress tolerance (including resistance to contamination and environmental fluctuations), and improved processability (e.g., self-flocculation, reduced cell-wall recalcitrance, and lower off-flavor production). Gene editing and metabolic engineering have already demonstrated substantial potential for enhancing biomass productivity and tailoring the composition of proteins, lipids, pigments, and other value-added compounds. However, the commercialization of genetically engineered microalgae for food applications requires robust safety assessment frameworks. Although laboratory and contained-cultivation studies have demonstrated the feasibility and relative genetic stability of several engineered strains, data on long-term toxicological safety, environmental persistence, horizontal gene transfer, ecological interactions, and unintended metabolic alterations remain limited. Therefore, comprehensive risk assessment and long-term monitoring are essential before large-scale food applications or environmental deployment of genetically engineered microalgae [134,135].

The circular economy model achieves a win-win for environmental and economic benefits. Integrating microalgae cultivation with wastewater treatment (industrial and agricultural wastewater) and CO_2_ fixation (industrial flue gas) enables a closed-loop model that closely links wastewater, microalgae, food, and byproduct utilization. Promoting microalgae-aquaculture-agriculture linkages, for example, treating aquaculture wastewater with microalgae and using the biomass as algal fertilizer, can significantly improve crop yield and quality. Nevertheless, such wastewater-grown microalgal biomass needs to comply with relevant agricultural and novel food regulatory requirements before formal field application.

## 6. Conclusions

Microalgae represent a high-quality and sustainable novel food resource with considerable nutritional, functional, and environmental potential. Significant advances have been achieved in strain discovery, cultivation technologies, bioprocess engineering, and value-added product development. Product formats are evolving from whole-biomass powders toward refined ingredients, bioactive extracts, and diversified food products, expanding opportunities across multiple food sectors. Nevertheless, the large-scale commercialization of microalgae-derived foods continues to face several interconnected challenges, including sensory limitations (off-flavor, color, and texture), high production and processing costs, and important scientific knowledge gaps related to nutrient bioavailability, long-term safety, and human health outcomes. In addition, regulatory and legislative challenges remain a critical bottleneck. Differences in approval procedures, safety assessment requirements, labeling standards, and market access regulations among jurisdictions can slow innovation, increase development costs, and limit international commercialization. Therefore, the establishment of science-based, transparent, and internationally harmonized regulatory frameworks, together with standardized methods for safety and quality evaluation, will be essential for the sustainable growth of the microalgae food sector.

Future development of the microalgae food industry will depend on the integration of advances in synthetic biology, food science, intelligent manufacturing, and sustainable bioprocessing, supported by close collaboration among academia, industry, and regulatory agencies. Priority research areas include the development of next-generation strains with improved productivity and functionality, optimization of sensory quality and consumer acceptance, reduction in production costs through low-carbon and automated processing systems, and generation of robust clinical evidence regarding nutritional efficacy and long-term safety. Equally important is the continued refinement and international harmonization of regulatory frameworks capable of addressing emerging technologies such as gene-edited strains, novel bioactive ingredients, and circular bioeconomy production systems. As technological innovation, scientific evidence, regulatory clarity, and consumer awareness continue to advance, microalgae-derived foods are increasingly likely to transition from niche products to mainstream components of sustainable food systems, contributing to global food security, environmental sustainability, and human health.

## Figures and Tables

**Table 1:** China-approved algae-related novel food ingredients.

Name	Basic Information	Intake	Applicable Food Categories	Announcement No.
*M. gaditana* oil	Genus: *Microchloropsis*	≤2 g/day	Not suitable for infants, pregnant women, or lactating women	No. 5, 2024
*Chlamydomonas reinhardtii*	Family: Chlamydomonadaceae; Genus: *Chlamydomonas*	≤10 g/day	Not to be used in infant foods	No. 2, 2022
*M. gaditana*	Family: Monodopsidaceae; Genus: *Microchloropsis*	≤2 g/day (as dry weight)	Not suitable for infants, pregnant women, or lactating women	No. 5, 2021
*Nostoc sphaeroides*	Phylum: Cyanobacteria; Family: Nostocaceae; Genus: *Nostoc*	≤3 g/day (as dry weight)	Not suitable for infants, pregnant women, or lactating women	No. 10, 2018
*Euglena gracilis*	Phylum: Euglenophyta; Order: *Euglenales*; Genus: *Euglena*	No specific limit	Not to be used in infant foods	No. 10, 2013
*Chlorella pyrenoidosa*	Phylum: Chlorophyta; Genus: *Chlorella*	≤20 g/day	Not to be used in infant foods	No. 19, 2012
*Haematococcus pluvialis*	Phylum: Chlorophyta; Order: *Volvocales*; Genus: *Haematococcus*	≤0.8 g/day	Not to be used in infant foods	No. 17, 2010
Docosahexaenoic acid (DHA) Algal Oil	Main component: DHA Sources: *Schizochytrium* sp., *Ulkenia amoeboida*, *Crypthecodinium cohnii*	≤300 mg/day (as pure DHA; for use in infant foods, comply with relevant standards)	Not to be used in infant foods	No. 3, 2010
*Dunaliella salina* (extract)	Phylum: Chlorophyta; Order: *Volvocales*; Genus: *Dunaliella*	≤15 mg/day (as β-carotene)	Not to be used in infant foods	No. 18, 2009

Data source: Government Service Platform of the National Health Commission of the People’s Republic of China (https://zwfw.nhc.gov.cn/).

**Table 2:** EU-approved microalgae species and related information.

Organism	Phyllum/	Food Product Form	Regulatory Basis in EU	Status
*Arthrospira* spp.	Cyanobacteria	Whole biomass (powder, tablets, food ingredient)	Significant consumption before 15 May 1997 (Novel Food Catalogue)	Traditional food
*Chlorella* spp.	Chlorophyta	Whole biomass (powder, tablets, food ingredient)	Significant consumption before 15 May 1997 (Novel Food Catalogue)	Traditional food
*Tetraselmis chuii*	Chlorophyta	Frozen and dried biomass	Union List of Novel Foods	Authorized novel food
*Odontella aurita*	Bacillariophyta	Dried biomass	Union List of Novel Foods	Authorized novel food
*Euglena gracilis*	Euglenozoa	Dried whole-cell biomass	Union List of Novel Foods	Authorized novel food
*Schizochytrium* spp.	Stramenophiles	DHA-rich oil	Union List of Novel Foods (product-specific authorizations)	Authorized novel food ingredient

Data source: EU Novel Food Catalogue (https://food.ec.europa.eu/safety/novel-food/novel-food-catalogue_en, accessed on 10 April 2026), EU Union List of Novel Foods (https://eur-lex.europa.eu/legal-content/EN/TXT/?uri=CELEX:32017R2470, accessed on 12 April 2026), and European Food Safety Authority (EFSA) website (http://www.efsa.europa.eu).

**Table 3:** Selected FDA GRAS notices for microalgal ingredients relevant to food applications in the US.

GRAS Notice No.	Ingredient (as Notified)	Source Organism/Strain	FDA Response	Intended Conditions of Use	Exposure/Use Limitations Reported in Notice
GRN 127	Spirulina (dried biomass)	*Arthrospira platensis*	FDA stated it had no questions regarding the notifier’s GRAS conclusion	Ingredient in foods including bars, powdered nutritional drink mixes, popcorn, salads and pasta	0.5–3 g per serving depending on food category
GRN 417	Dried biomass of spirulina	*Arthrospira platensis*	FDA stated it had no questions regarding the notifier’s GRAS conclusion	Ingredient in beverages, breakfast cereals, dairy desserts, grain products, snack foods, soups and other specified food categories	0.5–3 g spirulina per serving
GRN 424	C-c-phycocyanin-enriched water extract of the cyanobacterium	*Arthrospira maxima* or *Arthrospira platensis*	FDA has no questions; some uses may require a color additive listing	As an ingredient in all foods except infant formulas and foods under USDA’s jurisdiction	At levels up to a maximum of 250 mg per serving
GRN 137	DHA-rich algal oil	*Schizochytrium* sp.	FDA stated it had no questions regarding the notifier’s GRAS conclusion	Source of DHA for use in a variety of conventional foods	Estimated dietary exposure evaluated within the notifier’s proposed use levels
GRN 580	*Haematococcus pluvialis* extract containing astaxanthin esters	*Haematococcus pluvialis*	FDA has no questions; some uses may require a color additive listing	For use as an ingredient in baked goods and baking mixes, beverages and beverage bases, energy, sports, and isotonic drinks, non-milk-based meal replacements, cereals and cereal products, chewing gums, coffee, tea, dairy product analogs, frozen dairy desserts and mixes, hard and soft candy, milk products, processed fruits and fruit juices, and processed vegetables and vegetable juices.	At a maximum level of 0.15 mg astaxanthin per serving
GRN 294	*Haematococcus pluvialis* extract containing astaxanthin esters	*Haematococcus pluvialis*	FDA has no questions; some uses may require a color additive listing	For use as an ingredient in baked goods, beverages, cereals, chewing gum, coffee and tea, dairy product analogs, frozen dairy desserts and mixes, hard candy, milk products, processed fruits and fruit juices, processed vegetables and vegetable juices, and soft candy.	At a use level to provide 0.1 mg of astaxanthin per serving
GRN 986	*Chlorella sorokiniana powder and C. sorokiniana micro powder*	*Chlorella sorokiniana*	FDA has no questions	For use as an ingredient in yeast breads, doughnuts, nutrition bars, protein and nutritional powders, pasta, and noodles	At levels ranging from 5 to 10 mg/g.
GRN 519	*Chlorella protothecoides* strain S106 flour with 40–75% protein	*Chlorella protothecoides*	FDA has no questions; some uses may require a color additive listing	For use as a source of protein in baked goods and mixes, breakfast cereals, meal replacements, cheeses, milk products, dairy and nondairy products, etc.	At a level intended to provide up to 5562 mg of the ingredient per day.
GRN 351	*Dunaliella bardawil*	*Dunaliella bardawil*	FDA has no questions; some uses may require a color additive listing	For use as ingredient in cheese, bread and rolls, mayonnaise, cookies, crackers, tofu, and soybean fermentation products	At a level of 100 mg per kilogram
GRN 621	Emulsified algal oil	DHA-containing oil derived from *Schizochytrium* sp.	FDA issued a response letter to the notifier; conditions of use are specified in the notice	Food ingredient for proposed food uses described in the GRAS dossier	Exposure assessment evaluated within the notifier’s intended use conditions
GRN 843	Algal oil containing 35% DHA	*Schizochytrium* sp. strain FCC-1324	FDA stated it had no questions regarding the notifier’s GRAS conclusion	Ingredient in food categories listed in 21 CFR 184.1472(a)(3)	When blended with other DHA/EPA sources: ≤1.5 g DHA/person/day and ≤3.0 g DHA + EPA/person/day combined

Data source: U.S. Food and Drug Administration (FDA). GRAS Notices Inventory. Available at: https://www.fda.gov/food/generally-recognized-safe-gras/gras-notice-inventory (accessed on 20 April 2026).

## Data Availability

No new data were created or analyzed in this study. Data sharing is not applicable to this article.
